# Changes in body pain among overweight and obese housewives living in Klang Valley, Malaysia: findings from the MyBFF@home study

**DOI:** 10.1186/s12905-018-0597-x

**Published:** 2018-07-19

**Authors:** Syafinaz Mohd Sallehuddin, Noor Safiza Mohamad Nor, Rashidah Ambak, Nur Shahida Abdul Aziz, Nor Azian Mohd Zaki, Mohd Azahadi Omar, Tahir Aris, Nur Salihah Nor Hissam, Saravanan A. L. Rajadurai, Nor Hafizah Ayob

**Affiliations:** 1 Institute for Public Health, National Institutes of Health, Ministry of Health Malaysia, Kuala Lumpur, Malaysia; 2 Kuala Lumpur General Hospital, Ministry of Health Malaysia, Jalan Pahang, Kuala Lumpur, Malaysia

**Keywords:** Visual analogue scale (VAS), Body pain, Overweight, Obese, Body mass index (BMI), Housewives

## Abstract

**Background:**

Obesity leads to the increase of pain at different parts of the body and it is a potential marker for complications of chronic diseases. This paper aims to assess changes in the body pain among overweight and obese housewives who participated in the My Body is Fit and Fabulous at home (MyBFF@home) study.

**Methods:**

Housewives aged 18 to 59 years old from the MyBFF@home study were selected and pain was measured using the Visual Analogue Scale (VAS) questionnaire. VAS measured the pain intensity at different parts of the body (score of 0–10). Data were collected at base line, 3 months and 6 months among the housewives in both the control and intervention group. Pain scores and other variables (age, Body Mass Index (BMI) and waist circumference) were analysed using SPSS version 22.

**Results:**

A total of 328 housewives completed the VAS questionnaires at baseline, while 185 (56.4%) of housewives completed the VAS at 3 months and 6 months. A decreasing trend of mean pain score in both groups after 6 months was observed. However, the intervention group showed a consistent decreasing trend of pain score mainly for back pain. In the control group, there was a slight increment of score in back pain from baseline towards the 6 months period. Older housewives in both groups (aged 50 years and above) had a higher mean score of leg pain (2.86, SD: 2.82) compared to the other age group. Higher BMI was significantly associated with pain score in both groups.

**Conclusion:**

There were some changes in the level of body pain among the housewives before and after the intervention. Older obese women had a higher pain score compared to younger obese women. Pain was associated with BMI and change in BMI appears to be beneficial in reducing body pain among overweight and obese individuals.

## Background

The International Association of Pain defined pain as “an unpleasant sensory and emotional experience associated with actual or potential tissue damage” [1]. Even though no cause-effect relationship has yet to be established, there is growing evidence of an association between obesity and musculoskeletal injury and pain [1, 2]. It is a major issue as chronic pain in obesity may lead to obesity-induced problems and the deterioration of fitness and health-related quality of life (HR-QOL) and its capacity [3, 4]. According to Zdziarski (2015) and Okifuji (2015) the relationship between obesity and body pain is affected by several factors including high sedentary lifestyle issues and lack of exercises, inflammatory mediators and psychological factors such as depression and sleep disturbance [4, 5]. It could also be caused by load increasing activity such as walking, running or stairs climbing [5].

Past studies have reported the association between obesity and pain in different parts of the human body. The most frequently reported were osteoarthritis (OA) (knee, hip, hand) and low back pain [2]. Overweight and obese individuals especially those with the Body Mass Index (BMI) ≥ 30 kg/m^2^ also reported to have musculoskeletal pain and low-back pain when performing daily routines and activities [4, 6–8]. These factors contributed to the fear of becoming more active in order to lose weight and to reduce the pain [9–11]. The National Institute for Clinical Excellence (2017) suggested that weight reduction programme for overweight and obese adults as a basic component of chronic pain management [12]. This can be supported through a previous study by McGoey et al. (1990) which stated that if an obese individual loses 6 to 10 kg of weight, there is an association with a relief of pain in the lower back, ankle and feet [13].

As chronic pain interfered with daily functioning of obese individuals, it can have a negative effect on weight loss [5, 9]. There are several measurements used in past studies to assess body pain which include the Visual Analogue Scales (VAS), the Numeric Rating Scale for Pain (NRS Pain), the McGill Pain Questionnaire (MPQ), the Chronic Pain Grade Scale (CPGS), the Short-Form 36 Bodily Pain Scale (SF-36 BPS) and the Measure of Intermittent and Constant Osteoarthritis Pain (ICOAP) [14]. These measurements were widely used in different settings to assess chronic pain related to specific diseases and the VAS is the most common method used in the clinical and the community settings. It is a practical and concise tool, which comes along with a helpful graphic diagram and is more suitable for various community groups. There is limited evidence on the utilisation of the VAS in a community-based weight loss intervention study in Malaysia. Therefore, the aim of this paper was to assess changes in body pain of overweight and obese women who participated in the MyBFF@home study.

## Methods

The My Body is Fit and Fabulous at home (MyBFF@home) study was a community-based weight loss intervention study among housewives in Klang Valley, Federal Territory of Kuala Lumpur. In the present study, data of 328 housewives who participated in the MyBFF@home were used (Intervention; 169, Control; 159). The details of the methodology and the baseline participants’ characteristics of the MyBFF@home were explained elsewhere [15].

### Body pain measurement

In the MyBFF@home study, different parts of body pain were measured using the VAS questionnaire. VAS is a practical way to measure the intensity of pain among the adults in the community [8, 16–18]. VAS is a visual, reliable, simple tool and suitable for various types of respondents. It is a single-item scale consisting of a horizontal or vertical line, usually 10 cm (100 mm) in length, secured by 2 verbal descriptors, one for each symptom extreme [17]. Instructions, time period for reporting and verbal descriptor anchors have varied widely in the literature depending on intended use of the scale [7]. For pain intensity, the scale is most commonly anchored by “no pain” (score of 0) and “pain as bad as it could be” or “worst imaginable pain” (score of 10 [100 mm]). The recall period for VAS items varies, but most commonly used recall period was “recent” experience of pain intensity or the occurrence of pain and its intensity “in the last 24 hours”. VAS has good test-retest reliability and good validity to measure pain intensity [17].

**Fig. 1:**
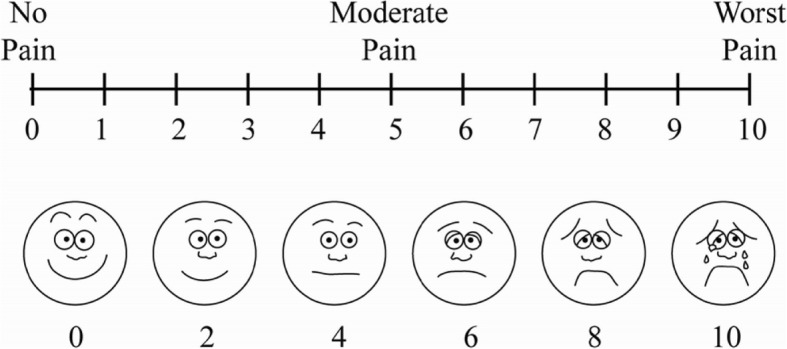
Graphic format of the VAS scale

In the MyBFF@home study, body pain was measured in four parts of the body: back bone, hand joints (e.g. finger, elbow, shoulder, arm, and wrist), leg joints (e.g. knee, ankle, toes and heel) and other areas (waist, hip, neck). Body pain assessments were performed among the control and the intervention groups at baseline (0 month), 3 month and 6 months follow up. The VAS interview was conducted by research officers with Nutrition and Dietetics qualifications using a standard questionnaire. During the interview, participants were given a graphic format of VAS (Fig. 1) to indicate the category of pain intensity level, which included no pain (0–4), moderate pain (5–7) and worst pain (8–10). Higher scores indicated greater pain intensity.

### Anthropometric measurement and socio demographic variables

Body weight and height of the participants were measured twice at baseline, 3 months and 6 months using the digital weighing scale (Tanita HD319, Japan) and SECA Bodymeter. The weight and height were measured to the nearest 0.1 kg and 0.1 cm. Waist circumference was also measured twice using the SECA measuring tape (SECA, Germany) with a range of measurement of 0–200 cm. The mean values of two measurements for all anthropometric data were used in the analysis. The socio demographic variables were age and BMI. The BMI was calculated based on the weight and height of the participants and then classified using the World Health Organisation (WHO),1998 classification for obesity. The World Health Organisation (WHO) BMI classifications were: underweight (BMI < 18.5 kg/m^2^), normal weight (18.5 to < 25 kg/m^2^) and overweight (≥ 25 kg/m^2^). Overweight is further subdivided into four categories: pre-obese (25 to < 30 kg/m^2^), obese Class I (30 to < 35 kg/m^2^), obese Class II (35 to < 40 kg/m^2^) and obese Class III (≥40 kg/m^2^) [18].

Data analyses involved descriptive statistics and paired t-tests to determine significant changes at baseline and post-intervention within each group. Repeated Measures ANOVA was performed and data were analysed using SPSS version 22 (SPSS Inc) for Windows. All statistical tests were considered significant at a *p* < 0.05 level.

## Results

A total of 328 housewives completed the VAS questionnaires at baseline and 185 respondents completed the VAS at 3 months and 6 months. In Table 1, based on age group, there were higher mean pain scores for the back bone and leg among 18–29-year-olds in both groups. Housewives aged more than 50 years old showed a higher mean pain score for the leg 2.86 (SD: 2.82) for both groups compared to other age groups. There was no significant difference in mean pain scores for the back bone and hand for both groups, however a significant difference (*p*-value < 0.05) was found in mean pain score for the leg based on Body Mass Index (BMI) categories. Based on waist circumference measurement, there was no significant difference in pain scores between housewives whose waist circumferences measured less than 80 cm and those more than 80 cm.

**Table 1:** Baseline mean pain score within intervention phase based on socio-demography characteristic

Characteristic/ Pain Scale		Back bone		Hand		Leg		Others	
	N	Mean(SD)	*p*-value	Mean(SD)	p-value	Mean(SD)	p-value	Mean(SD)	*p*-value
Intervention group (n:168)									
Age group:									
18–29	14	2.14(3.11)	0.286	1.14(2.14)	0.859	4.07(4.01)	0.162	0.93(2.23)	0.963
30–39	47	1.47(2.38)		1.29(2.19)		2.21(2.69)		1.06(2.06)	
40–49	71	0.99(2.17)		0.96(2.01)		2.42(2.71)		1.01(2.15)	
> 50	35	0.97(2.26)		1.06(1.99)		2.86(2.82)		1.23(2.46)	
BMI category:									
25.0–29.9	73	1.04(2.25)	0.676	1.12(2.05)	0.472	1.97(2.83)	0.014	1.10(2.26)	0.710
30.0–34.9	58	1.26(2.29)		0.84(1.84)		2.86(2.80)		1.12(2.19)	
35.0–39.9	32	1.20(2.35)		1.38(2.22)		2.63(2.89)		0.76(1.94)	
Waist circumference									
< 80.0 cm	10	1.90(2.47)	0.342	1.90(2.47)	0.200	2.90(3.18)	0.723	1.90(2.72)	0.214
> 80.0 cm	158	1.17(2.34)		1.04(2.02)		2.57(2.86)		1.01(2.14)	
Control group(n:159)									
Age group:									
18–29	14	1.5(2.24)	0.886	0.8(2.00)	0.941	1.1(2.18)	0.016	1.6(2.44)	0.702
30–39	48	1.3(2.38)		1.0(2.27)		2.3(2.66)		1.5(2.47)	
40–49	66	1.4(2.12)		0.8(1.85)		1.8(2.84		1.2(2.22)	
> 50	31	1.0(1.91		1.0(1.88)		3.6(3.25)		3.6(3.25)	
BMI category:									
25.0–29.9	78	1.4(2.13)	0.476	0.9(1.86)	0.872	2.0(2.63)	0.399	1.2(2.00)	0.484
30.0–34.9	48	1.4(2.38)		1.0(2.06)		2.3(2.86)		1.8(2.88)	
35.0–39.9	30	0.8(1.76)		0.8(2.32)		2.8(3.56)		1.6(2.55)	
Waist circumference									
< 80.0 cm	10	1.4(2.27)	0.885	0.5(1.08)	0.509	1.2(2.15)	0.236	0.8(1.68)	0.351
> 80.0 cm	148	1.3(2.16)		0.9(2.04)		2.3(2.93)		1.5(2.46)	

*Significant value at, p-value < 0.05

Table 2 shows pain category in four parts of the body at baseline. Most respondents in the intervention group reported no pain at all. Some of them reported that their leg pain was in the moderate pain category (33.7%) and a few in the severe category (8.7%). The same situation was seen in the control group where they reported leg pain in the moderate category (27.7%). Most of the housewives were in obesity Class I category, which explains the leg pain while walking.

**Table 2:** Pain category between groups in intervention phase

	Intervention (n:169)								Control (159)							
Pain Category	No pain		Mild		Moderate		Severe		No pain		Mild		Moderate		Severe	
	n	%	n	%	n	%	n	%	n	%	n	%	n	%	n	%
Back bone	126	74.6	11	6.5	23	13.6	8	4.7	110	69.2	14	8.8	32	20.1	3	1.9
Hand Joint (finger, elbow, shoulder, wrist)	123	72.8	16	9.5	23	13.6	6	3.6	127	79.9	9	5.7	21	13.2	2	1.3
Leg joint (hamstring, knee, ankle, toes and heel)	83	49.1	15	8.9	57	33.7	14	8.3	87	54.7	16	10.1	44	27.7	12	7.5
Others (waist, hip, neck)	131	77.5	8	4.7	22	13.0	7	4.1	106	66.7	16	10.1	30	18.9	7	4.4

**Fig. 2:**
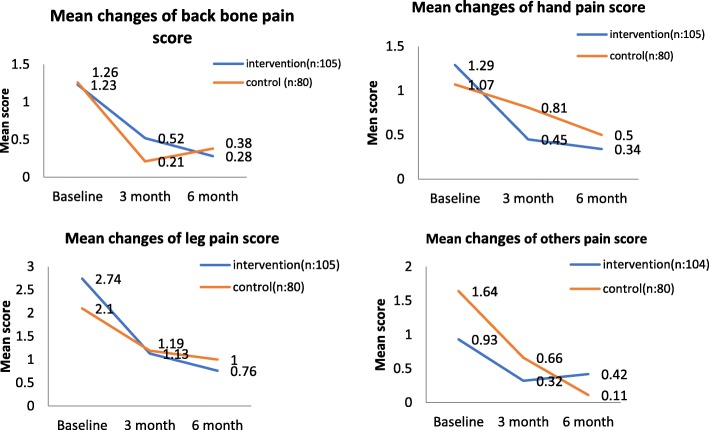
Mean changes of pain score between group in intervention phase

Figure 2 shows a decreasing trend in pain intensity in all body parts for both groups after 6 months of the intervention programme. The intervention group showed a consistent decreasing trend of mean pain score in the back bone, with a mean difference of 0.95 between baseline and 6 months, and for the control group there was a slight increment when approaching 6 months of intervention. However, mean pain scores in the hand and leg showed a decreasing trend for both groups. The intervention group showed a slightly increasing trend of mean pain score in other body parts (waist, hip and neck), with a mean difference between baseline and 6 months of 0.51.

A two-way repeated measures ANOVA (RM-ANOVA) was conducted to compare the effect of time of intervention baseline to 6 months on pain score changes before, during, and after the intervention, as shown in Table 3. There was a significant effect of time of intervention on pain score changes, Wilks’ Lambda = 0.84, F (2,182) =16.80, p = < 0.001. As indicated from RM-ANOVA, there was no significant difference between both groups for back bone, hand, leg and others (*p* > 0.05). Although there was a decrease in mean pain score, there was no significant difference in intervention effects for back bone, hand and leg pain. Only other parts of the body showed a significant difference in intervention effect (*p* < 0.05).

**Table 3:** Changes in pain score using repeated measure ANOVA

Variable	Group	N	Mean difference (Baseline- 6 month)	*p*-value		
				Time	Group	Intervention effect
Back Bone	Intervention	105	0.95	0.005*	0.733	0.401
	Control	80	0.88	0.003*		
Hand	Intervention	105	0.57	0.001*	0.599	0.219
	Control	80	0,94	0.167		
Leg	Intervention	105	1.98	0.001*	0.660	0.092
	Control	80	1.10	0.002*		
Others	Intervention	105	0.51	0.115	0.160	0.009*
	Control	80	1.1	0.002*		

Time effect - Repeated Measure ANOVA within group analysis was applied

Mauchly’s test of Sphericity was done (*p*-value < 0.001)

*significant at p < 0.05

## Discussion

Our study examined how pain was described in this community-based weight loss intervention and the results are in line with the previous study [16, 19]. The results showed an increment in mean pain score at the backbone and leg area among the overweight and obese women. The level of pain and number of painful areas also increased with obesity level. Pain could also be compounded by increased BMI [20] and those experiencing chronic pain slowed down their routines and had a declined in strength and mobility [16, 19, 20].

Excess weight is said to be associated with increases in the amount of force on a weight bearing joint, and there is also a positive relationship between BMI and knee OA [2, 5]. There is also an association between weight increase and shoulder pain, heel pain and hip pain among middle age and elderly individuals [2, 3, 5]. However, other studies on subjects with hip OA have not found BMI to be a risk factor [3, 6]. These studies reported either hip or knee pain, as well as ankle pain [3]. In another study of patients with knee pain whose knee OA was not known, there was a positive association between BMI and pain scores [6].

Pain category in adults is complex as many people have pain of varying intensity and duration in more than one body region. For the present study, pain was assessed according to location and severity of pain at a number of musculoskeletal regions. Respondents were interviewed about pain they feel at different body parts and were asked to rate their pain level according to pain rating scale VAS. In our study however, findings showed the respondents in type 1 obesity have leg pain while walking which was common, however the score was not significant in either the intervention or control group [10, 20].

In a previous study carried out among older population in a western setting, it was found that widespread musculoskeletal pain, the most extensive pain category, was defined as pain in the upper joints (hand or wrist) and lower joints (hip, knee or foot) and axial skeletal pain (back or chest) [19]. Another study found that chronic muscular pain of lower extremities or joints is usually found in women [20]. Most studies done, including our study had mentioned that pain classification was determined at baseline and at each follow up round. [6].

In changes of mean score, there were no significant differences between the groups [11]. However, there were some changes found, which showed a decrease in pain score for hand and in others in the intervention group [8, 11]. In another study, among western post bariatric surgery patients, there was a significant decrease in pain at most sites following weight loss and physical exercise after 6 to 12 months post intervention, especially in the cervical and lumbar spine, and foot [2].

The strength of this study included the combination intervention package consisted of dietary, self-monitoring, physical activity and the screening of VAS, which was one of the acceptable and reliable measure of pain intensity [17]. This survey had certain limitations such as the possible under or over-reporting of pain ratings by the housewives which may contribute to the bias of the results, hence the further probing by the interviewer during data collection session to confirm the housewives’ responses to try to minimise the bias. Since our study population consisted of urban housewives, our finding may not be generalized to all housewives in Malaysia.

## Conclusion

In conclusion, body pain is associated with BMI. There were changes in the level of pain before and after the intervention. Obese women and older women had higher pain scores compared to overweight and younger women. The results of this study indicated there was a weak risk for body pain as weight increases, therefore overweight and obese persons must be considered as a high-risk group. This indicates the need for precaution and prevention measures of weight control. Future research focusing on prevention of obesity and maintaining a healthy weight is warranted to help lower the risk of pain in body parts that could be caused by obesity or overweight.

## Abbreviations

BMI: Body mass index
CPGS: Chronic pain grade scale
ICOAP: Intermittent and constant osteoarthritis pain measurement
MPQ: The McGill pain questionnaire
MyBFF: My body fit fabulous
NRS Pain: Numeric rating scale for pain
RM-ANOVA: Repeated measures - analysis of variance
SF-36 BPS: Short-Form 36 bodily pain scale
VAS: Visual analogue scale
